# Molecular Precursor Routes for Ag-Based Metallic, Intermetallic, and Metal Sulfide Nanoparticles: Their Comparative ORR Activity Trend at Solid|Liquid and Liquid|Liquid Interfaces

**DOI:** 10.1021/acs.inorgchem.3c00978

**Published:** 2023-05-16

**Authors:** Malik Dilshad Khan, Magdalena Warczak, Ginena Bildard Shombe, Neerish Revaprasadu, Marcin Opallo

**Affiliations:** † Institute of Physical Chemistry, Polish Academy of Sciences, Kasprzaka 44/52, Warsaw 01-224, Poland; ‡ Department of Food Analysis and Environmental Protection, Faculty of Chemical Technology and Engineering, Bydgoszcz University of Science and Technology, Seminaryjna 3, Bydgoszcz 85-326, Poland; § Chemistry Department, University of Dar-es-Salaam, P.O. Box 35061, Dar-es-Salaam 63728, Tanzania; ∥ Department of Chemistry, University of Zululand, Private bag X1001, Kwa-Dlangezwa 3880, South Africa

## Abstract

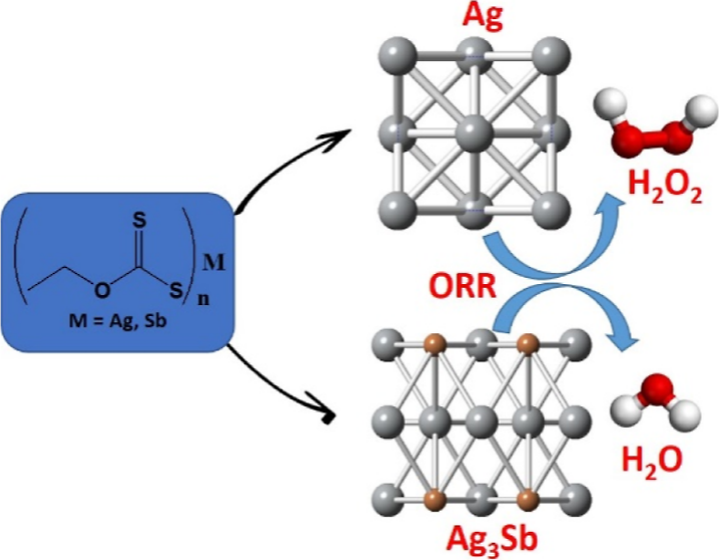

The electrochemical conversion of oxygen to water is a crucial process required for renewable energy production, whereas its first two-electron step produces a versatile chemical and oxidant—hydrogen peroxide. Improving performance and widening the limited selection of the potential catalysts for this reaction is a step toward the implementation of clean-energy technologies. As silver is known as one of the most effective catalysts of oxygen reduction reaction (ORR), we have designed a suitable molecular precursor pathway for the selective synthesis of metallic (Ag), intermetallic (Ag_3_Sb), and binary or ternary metal sulfide (Ag_2_S and AgSbS_2_) nanomaterials by judicious control of reaction conditions. The decomposition of xanthate precursors under different reaction conditions in colloidal synthesis indicates that carbon–sulfur bond cleavage yields the respective metal sulfide nanomaterials. This is not the case in the presence of trioctylphosphine when the metal–sulfur bond is broken. The synthesized nanomaterials were applied as catalysts of oxygen reduction at the liquid–liquid and solid–liquid interfaces. Ag exhibits the best performance for electrochemical oxygen reduction, whereas the electrocatalytic performance of Ag and Ag_3_Sb is comparable for peroxide reduction in an alkaline medium. Scanning electrochemical microscopy (SECM) analysis indicates that a flexible 2-electron to 4-electron ORR pathway has been achieved by transforming metallic Ag into intermetallic Ag_3_Sb.

## Abstract 2

Xanthate complexes of Ag and Sb were used to selectively prepare metallic, intermetallic, and metal sulfide nanomaterials. The oxygen reduction reaction can be tuned from a 2-electron to a 4-electron mechanism by transforming Ag into Ag_3_Sb.

## Introduction

Renewable energy sources, such as metal–air batteries and fuel cells, require suitable electrocatalysts with high activity for oxygen reduction reactions (ORRs).^1^ Electrochemical oxygen reduction follows a 2- or 4-electron pathway leading to the formation of H_2_O_2_ or H_2_O, respectively.^2^ Both reactions are of immense importance because H_2_O_2_ is a versatile industrial chemical and a benign oxidant, whereas 4-electron reduction is preferred for low-temperature fuel cells.^3^ In addition, H_2_O_2_ is a promising green energy source as it releases 96 kJ mol^–1^ of energy with only H_2_O and O_2_ as products.^4^ The chemical energy can be converted into electrical energy via fuel cells.^5^ Therefore, robust catalysts for oxygen reduction or H_2_O_2_ reduction are vital as renewable and sustainable materials.

Platinum and platinum-based materials exhibit remarkable catalytic activity toward ORR.^6^ However, their rare occurrence and extremely high cost is a major hurdle to their commercial use on a large scale. Alternatively, silver (about 50 times less expensive than Pt) exhibits promising electrocatalytic activity and stability for ORR in alkaline media.^7^ The ORR at Ag, similarly as that at Pt, involves adsorption of O_2_ at the metal surface.^8^ However, the relatively poor affinity of O_2_ to Ag makes it difficult to break the O–O bond, which accounts for the lower catalytic activity as compared to Pt.^9^ Therefore, it is desirable to search among silver compounds to strengthen Ag–O interactions. It is known that the reactivity of electrocatalysts can be tailored by the modification of the adsorption energies of reaction intermediates.^10^ Some approaches to alter the adsorption energies are based on controlling the shape and size of the electrocatalysts. In this way, catalytic activity may be enhanced only to a certain extent because the nature of the material remains the same.^11^ In contrast, extensive research has been dedicated to engineering the structure and composition (i.e*.*, combined with other elements to form alloys or intermetallic compounds) of the electrocatalysts to enhance the catalytic activity.^12^ Besides ORR, silver and its compounds are considered as effective catalysts of H_2_O_2_ reduction as well.^13^

Among the strategies to obtain effective silver-based catalysts, the formation of binary or ternary alloys results in substantial electronic modulation and better performance due to synergistic effects and regulation of oxygen adsorption energies from different elements.^12a^ The order of activity of Ag-based alloys of type Ag_75_M_25_ (where M = Cu, Co, Fe, and In) toward ORR in alkaline media is as follows: Ag_75_Cu_25_ > Ag_75_Fe_25_ > Ag_75_Co_25_ > Ag > Ag_75_In_25_.^12a^ Other studies also indicated that the nature and concentration of the incorporated metal significantly affect their catalytic performance.^14^ Although silver-based alloys were studied extensively, only a few intermetallic compounds of silver were applied as ORR catalysts. For example, alloying oxophilic Sn produces Ag_3_Sn nanoparticles that exhibit significantly better performance for both ORR and borohydride oxidation reactions than pure Ag nanoparticles.^15^ Similarly, Ag_4_Sn nanoparticles dispersed on carbon showed superior ORR activity in a direct methanol alkaline fuel cell as compared to Ag nanoparticles.^16^

While the ORR at metallic Ag is well explored,^7^ activity of its ionic form was not well studied. Recently, the active nature of ionic silver toward ORR in an alkaline medium was reported.^17^ The Ag-based molecular organic framework was prepared from benzene tricarboxylic acid, and this coordination compound showed promising ORR electrocatalysis with a relatively much smaller loading of silver as compared to other Ag-based catalysts.^17^ This study advocates research on catalytic centers besides a few platinum group metal-free catalytic sites.

The synthesis of silver-based alloys, intermetallics, or ternary compounds requires judicious control of reaction parameters to obtain the precise composition of the synthesized materials. In this respect, metal–organic precursors are advantageous due to the presence of preformed bonds between the metal and the chalcogenide atoms.^18^ Moreover, these precursors are highly versatile, stable, less toxic, and easy to handle under normal conditions.^19^ The metal–organic precursors have not been explored for the synthesis of intermetallic compounds.

Moreover, unlike Pt-based alloys, the structure–activity relationship of Ag-based compounds was not well studied. In order to identify and understand the relationship between differences in electronic structures induced by a combination with other elements and ORR activity, different Ag-based nanomaterials were selected for this study. To diversify the nature of samples, different combinations of elements were used in such a way that Ag is combined with another metal to form an intermetallic compound (Ag_3_Sb), a non-metal to form a binary chalcogenide (Ag_2_S), and a combination of both these elements (metal and non-metal) to form a ternary chalcogenide of silver, i.e., AgSbS_2_. The systematic study of these materials can provide a basic idea regarding the suitable combinations of Ag with other elements for further explorations. They were synthesized by the hot injection method using metal–organic precursors. To the best of our knowledge, this is the first example of the synthesis of an intermetallic compound using sulfur-based metal–organic precursors. A plausible mechanism involving the role of trioctylphosphine (TOP) in the formation of metallic or intermetallic compounds is proposed. The electrochemical behavior of synthesized Ag-based electrocatalysts immobilized on the electrode surface within the ionomer film with respect to ORR and H_2_O_2_ reduction was investigated and discussed in terms of their structure and composition. We will also demonstrate that these materials can be assembled at a liquid–liquid interface. Such an almost molecularly flat self-healing interface allows studying of nanomaterials in the absence of a solid surface or binder.^20^ Until now, only noble metal nanoparticles (Au, Pt, and Pd) were applied to ORR at a liquid|liquid interface.^21^ Besides noble metals, our group has previously investigated the ORR performance of MoS_2_^22^ and Li-ion battery waste material^23^ at a liquid|liquid interface.

## Experimental Section

### Materials and Methods

Potassium ethyl xanthogenate, silver nitrate, antimony chloride, decamethylferrocene (DMFc), KOH (99.9%), HClO_4_, α,α,α-trifluorotoluene (TFT) (anhydrous, ≥99%), oleyl amine (OLA), TOP, 1-octadecene (ODE), Nafion (∼5% in a mixture of lower aliphatic alcohols and water), and ethanol were purchased from Sigma-Aldrich and were used as received. Argon gas was supplied by Multax (99.999% purity). Water was purified with an Arium Sartorius purification system and stored in glass. (Note: The chemicals used are corrosive to the eyes and skin, flammable, and toxic to aquatic life. Therefore, they should be handled carefully in a fume hood using proper PPE, and residual chemicals should be disposed of properly.)

### Synthesis of *tris*(O-Ethyldithiocarbonate)antimony(III) Complex

Antimony ethyl xanthate complex was synthesized by a previously reported method.^24^ Briefly, an ethanolic solution (25.0 mL) of SbCl_3_ (2.3 g, 10.0 mmol) was added slowly to an ethanolic solution (40 mL) of potassium ethyl xanthate (4.8 g, 30.0 mmol) while stirring. After a while, yellow-colored precipitates were formed, which were filtered, washed with water, dried, and recrystallized from chloroform. Yield (81%); anal. for C_9_H_15_O_3_S_6_Sb, found (%), C, 22.61; H, 3.07; S, 39.71; Sb, 24.15; calc. (%) C, 22.25; H, 3.11; S, 39.55; Sb, 25.08.

### Synthesis of (O-Ethyldithiocarbonate)Silver(I) Complex

The (O-ethyldithiocarbonate)silver(I) complex was synthesized by using a similar procedure, except that AgNO_3_ (5.3 g, 31.2 mmol) was used instead of SbCl_3_ salt. Although the addition of AgNO_3_ instantly yielded a light green-colored precipitate, the stirring was continued for a further half an hour to complete the reaction. The precipitate was filtered, washed with deionized water and ethanol, and dried in a desiccator without recrystallization, as the complex was insoluble in most organic solvents. Yield (5.2 g, 77%); anal. (calc.) for C_3_H_5_OS_2_Ag, C, 15.71; H, 2.20; S, 27.93; Ag, 47.1%; found, C, 15.82; H, 2.25; S, 28.2; Ag, 47.3%.

### Synthesis of Elemental (Ag, Sb) Nanoparticles

Metallic Ag or Sb nanoparticles were synthesized by the hot injection method. Briefly, 8.0 g, 30.0 mmol of OLA was placed in a three-necked round-bottom flask and heated to 120 °C. A vacuum was applied to degas and remove water and low boiling impurities. The flask was flushed with nitrogen and the temperature was raised to 200 °C and maintained there for 15 min. Once the temperature was stabilized, the respective xanthate complex of Ag or Sb (0.3 g) was dispersed in 3.0 mL of TOP and sonicated for a few minutes for uniform dispersion. The dispersed precursor was then immediately injected into preheated OLA at 200 °C. The temperature dropped by almost 15–20 °C but was readjusted to 200 °C again. The reaction was continued for 1 h, after which the heating was turned off. The flask was allowed to cool down and then a (30.0 mL) mixture of acetone and methanol (1:1) was added to stop the reaction. The precipitates were washed and separated from excess solvent/capping agents by centrifugation and dried for further analysis.

### Synthesis of Bimetallic Ag_3_Sb Alloy

For the synthesis of intermetallic Ag_3_Sb alloy, Ag and Sb xanthate complexes were properly mixed in a 3:1 molar ratio, respectively, and the powdered mixture was dispersed in (3.0 mL) TOP. It was sonicated for uniform dispersion and then injected into preheated OLA at 200 °C. Upon injection, the solution turned black immediately and a slight drop in temperature (*c.a.* 10–15 °C) was observed. The temperature of the reaction mixture was readjusted to 200 °C and then stirred for an hour. Then, the reaction mixture was allowed to cool down to room temperature. The precipitated product was washed and separated by centrifugation using a (30.0 mL) mixture of acetone and methanol. The black powdered product was dried at ambient conditions and used for further analysis.

### Synthesis of Binary Metal Sulfide (Ag_2_S, Sb_2_S_3_) Nanoparticles

Binary metal sulfide (Ag_2_S, Sb_2_S_3_) nanostructures were synthesized by thermal decomposition of respective xanthate complexes of silver and antimony (0.2 g) in OLA (6.0 mL). The procedure was similar to the synthesis of metallic nanoparticles, except for using ODE (3.0 mL) instead of TOP as the dispersion medium. All reactions were performed for 1 h and acetone/methanol (1:1) mixture was used to precipitate nanoparticles. The nanoparticles were washed properly to remove the extra capping agent and were dried in a desiccator for further analysis.

### Synthesis of Ternary AgSbS_2_ Nanoparticles

Ternary metal sulfide (AgSbS_2_) nanostructures were synthesized by thermal decomposition of equimolar 1:1 mixture of respective xanthate complexes of silver and antimony (0.5 mmol) in 6.0 mL of OLA. The procedure was similar to the synthesis of binary metal sulfide nanoparticles, using ODE (3.0 mL) as the dispersion medium. All reactions were performed for 1 h and acetone and methanol (1:1) mixture was used for the precipitation of the product. The nanoparticles were washed properly to remove the extra capping agent and dried for further analysis.

## Characterization

The elemental composition of synthesized complexes was obtained on an automated PerkinElmer 2400 series analyzer. Thermogravimetric analyses (TGA) were performed employing a Mettler-Toledo TGA/DSC. X-ray diffraction (XRD) was performed using a Bruker AXS D8 diffractometer in a 2θ range from 10 to 70°. The morphology of the samples was characterized by transmission electron microscopy (TEM) (JEOL 1400) with an accelerating voltage of 100 kV. The ultraviolet–visible (UV–vis) spectrum was recorded using a PerkinElmer Lambda 1050 instrument, using quartz cuvettes with a path length of 1 cm. A Bruker FTIR Tensor 27 spectrophotometer (wavenumber range of 450–4000 cm^–1^) equipped with a standard ATR crystal cell detector was used for infrared (IR) analysis.

### Electrochemical Experiments

Voltammetric characterization was performed with an Autolab potentiostat, employing a three-electrode system. Approximately, 5 mg of the synthesized nanomaterials were dispersed in 0.1 mL of Nafion and sonicated to form a homogenous suspension. The working GC disc electrode (0.0078 cm^2^) was modified by drop casting 2 μL dispersion of synthesized electrocatalysts in Nafion solution using a micropipette. This electrode material was selected because it hardly exhibits electrocatalytic properties. Platinum wire and Hg/HgO were used as counter and reference electrodes, respectively. All electrolyte solutions were prepared using ultrapure water from an Arium Sartorius (Millipore) purification system and stored in glass. Cyclic voltammograms (CV) were taken both in an Ar-purged and O_2_-saturated electrolyte solution. When necessary, argon gas was purged.

Scanning electrochemical microscopy (SECM) was performed with an Ivium Bipotentiostat (Ivium Technologies, Netherlands) in the three-electrode system. Pt microelectrodes, *c.a.* 25 μm diameter (Goodfellow, England) embedded in glass with the help of a capillary puller, were immersed in the aqueous phase and were used as SECM probes. A silver wire served as a pseudo-reference electrode to avoid the contribution of chloride-ion oxidation to the measured current.

### Thermogravimetric Analysis

The thermal stability of the metal (Ag, Sb) complexes in the solid state was studied by thermogravimetry in the range from 30 to 500 °C at a 10 mL min^–1^ flow rate under nitrogen. Both complexes undergo a single-step decomposition. The antimony xanthate complex decomposes at comparatively lower temperatures (145–155 °C) than the silver xanthate complex (170–175 °C) (Figure S1). A marginally small weight loss slightly below 250 °C for Sb xanthate may be attributed to an escape or volatilization of the residual product, which may slightly decrease the residual mass. Often at higher temperatures, metal chalcogenides are chalcogen deficient due to the high partial pressure of chalcogens.^25^ The residual masses obtained after the decomposition of the complexes indicate the formation of metal sulfides (Ag_2_S and Sb_2_S_3_). In order to further confirm the nature of the decomposition product, the solid-state decomposition of respective xanthate precursors was also carried out under inert conditions in a tube furnace, and the *p*-XRD pattern of the products also confirms the formation of respective metal sulfides (Figure S2).

## Results and Discussion

Xanthate complexes have been used extensively for the synthesis of different metal sulfide nanoparticles and thin films.^24b,26^ The O–R group of xanthate is very weakly electron donating. It lacks the extra electron density on sulfur atoms due to the absence of π donation of oxygen’s lone electron pair into the π electron system of CS_2_.^27^ For the same reason, xanthate complexes are usually less stable than other metal–organic precursors with similar chelating groups, such as dithiocarbamates or dithiophosphinates, and decompose at relatively lower temperatures. In addition, xanthate complexes were selected for this study because their decomposition byproducts are volatile, thereby leaving behind crystalline nanomaterials with high purity.

Initially, OLA was attempted to be used as a dispersion medium and capping agent for the synthesis of the respective metal sulfide nanomaterials. However, when the xanthate complexes are dispersed in OLA, the solution immediately turns black, indicating their decomposition even at room temperature. In order to investigate the nature of the decomposition products, the xanthate complexes of Ag and Sb were stirred in oleylamine at room temperature for 30 min and, afterward, the products were washed and isolated by centrifugation. *p*-XRD analysis indicates the formation of Ag_2_S nanoparticles by decomposition of Ag xanthate complexes even at room temperature, whereas decomposition of Sb xanthate yielded amorphous Sb_2_S_3_ (Figure S3). This is because primary amines are strong Lewis bases and can initiate the decomposition of xanthate complexes by attacking the thiocarbonyl center.^28^ Therefore, TOP or ODE was used as a dispersion medium. Because the decomposition of xanthate precursors in OLA at room temperature showed decomposition of amorphous products in the case of Sb xanthate (Figure S3), therefore, to obtain nanomaterials with high crystallinity and well-defined diffraction peaks, the complexes dispersed in ODE or TOP were injected into the preheated OLA at 200 °C.

**Figure 1:**
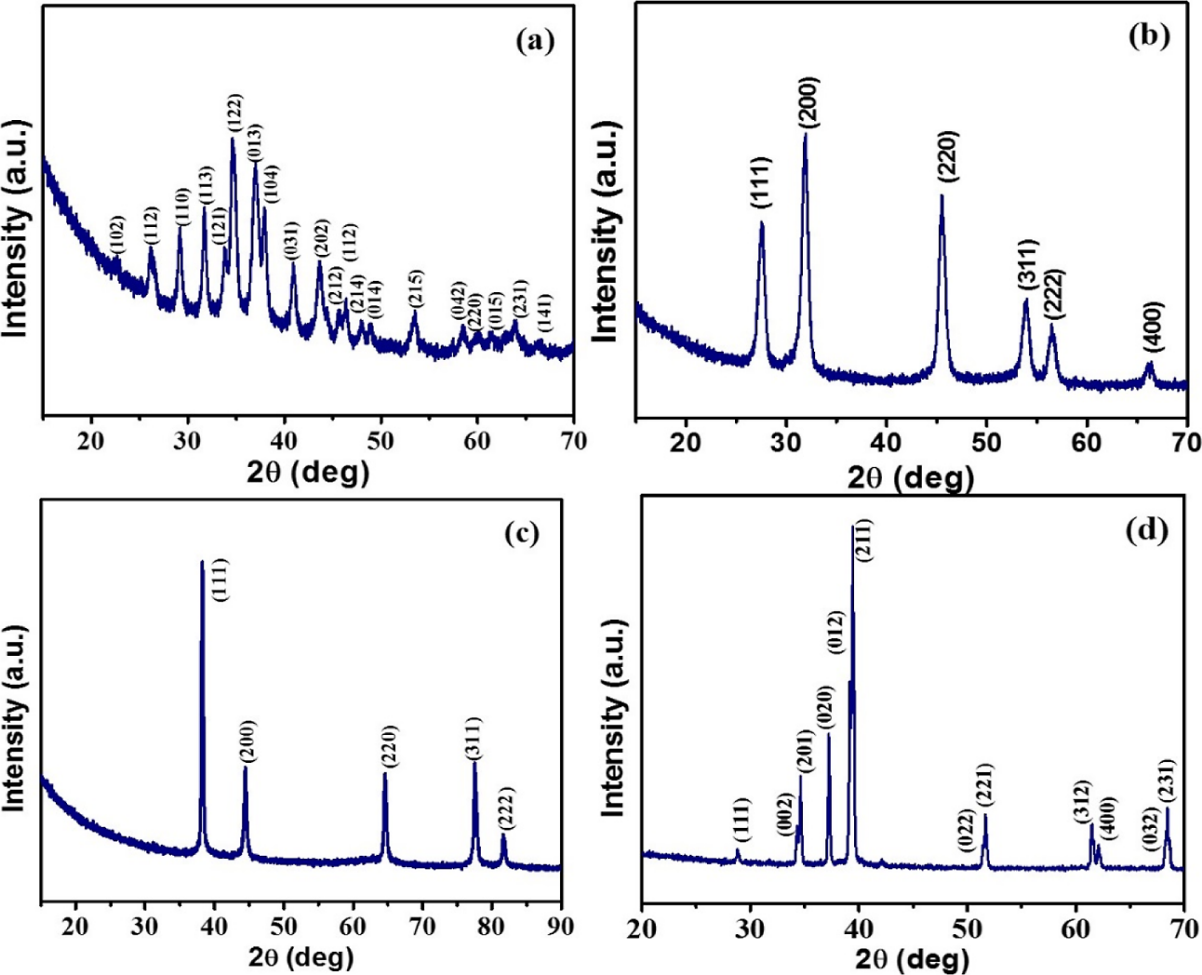
*p*-XRD analysis of (a) Ag_2_S, (b) AgSbS_2_, (c) Ag, and (d) Ag_3_Sb nanomaterials synthesized in oleylamine at 200 °C.

**Figure 2:**
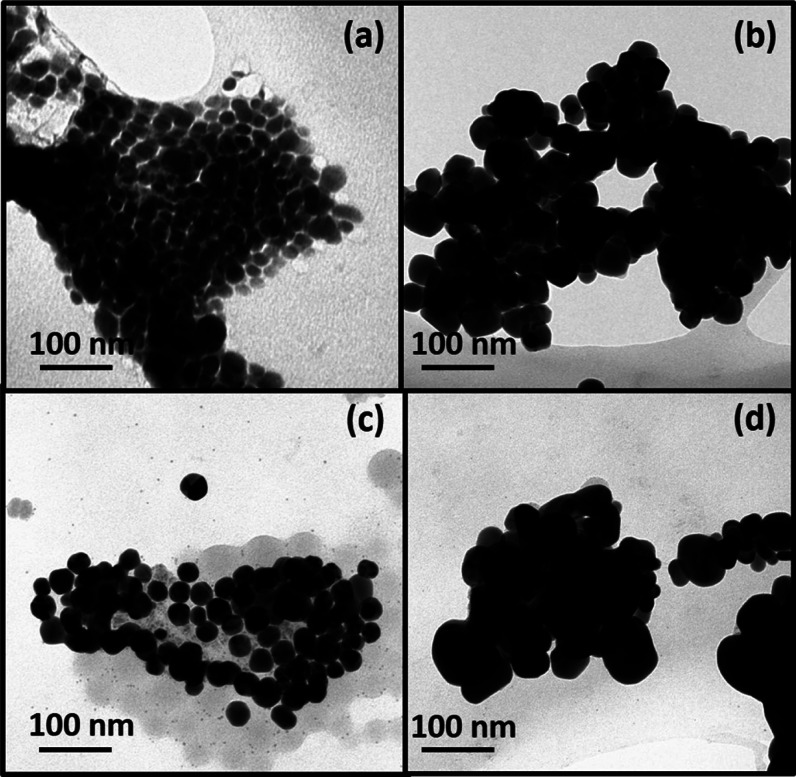
TEM images of (a) Ag_2_S, (b) AgSbS_2_, (c) Ag, and (d) Ag_3_Sb nanomaterials synthesized in oleylamine at 200 °C.

The *p*-XRD analysis indicates the formation of phase-pure Ag_2_S (ICDD # 00-024-0715) from the decomposition of xanthate precursors when dispersed in ODE. The diffraction pattern shows well-defined peaks with reasonable intensity (Figure 1a). Here, ODE acts as a dispersion medium and does not play any significant role in the decomposition of xanthate complexes or capping of the nanoparticles formed due to the lack of coordinating functional groups. Hence, only OLA is responsible for the decomposition of the complex and its shape and size control. TEM images indicate the formation of spherically shaped nanoparticles (Figure 2a), showing a broad size distribution (Figure S4). This is understandable on the basis of hard and soft acid and base concepts^29^ because the amine group of OLA is hard and silver is soft; therefore, in this case, OLA is not an effective capping agent.

Likewise, the decomposition of the antimony xanthate complex under similar reaction conditions resulted in the formation of the orthorhombic stibnite phase (ICDD# 01-075-1310) (Figure S5). Sharp and intense diffraction peaks indicate the high crystallinity of the synthesized Sb_2_S_3_ nanoparticles. The formation of Sb_2_S_3_ nanorods *via* the decomposition of xanthate precursor was previously reported in the literature.^30^

Apart from binary metal sulfides, the ternary compound AgSbS_2_ was prepared by dispersing equimolar (1:1) amounts of silver and antimony xanthate complexes in ODE with the help of sonication. This uniform dispersion was immediately injected into preheated OLA at 200 °C. The *p*-XRD analysis indicates the formation of the cubic AgSbS_2_ phase, and the diffraction peaks match well with the standard pattern (ICDD # 01-089-3669) (Figure 1b). As it is seen that the individual complexes decompose into their respective metal sulfides, however, no extra peak was observed for Ag_2_S, Sb_2_S_3_ or any other phase as an impurity. The peaks were slightly broad and intense, which is a signature of the formation of small-sized nanoparticles with good crystallinity. The size and morphology of synthesized nanoparticles were analyzed by TEM (Figure 2b). The nanoparticles were larger as compared to those of Ag_2_S. Their shape is far from symmetric, and a broad size distribution of nanoparticles was noted (Figure S4).

The transformation of metal–organic precursor into metallic Ag nanoparticles was performed by following similar reaction conditions, except that ODE was replaced by TOP (3.0 mL). Interestingly, the introduction of TOP resulted in the formation of pure silver from the xanthate complex, despite the fact that the silver complex showed higher stability by TGA analysis, and Ag and S atoms of the complex are directly bonded.^31^ The *p*-XRD analysis indicates the formation of highly crystalline elemental silver and there was no indication of Ag_2_S impurity (Figure 1c). The TEM analysis shows that Ag nanoparticles are spherical with a uniform size and shape (Figures 2c and S4).

When the complexes were dispersed in TOP, the suspension started to darken, which may indicate their decomposition initiation, even at room temperature. The interaction of TOP with metal complexes was examined by UV–vis spectroscopy. Antimony xanthate is well soluble in most organic solvents, whereas silver ethyl xanthate is not. Therefore, only the antimony complex was examined in chloroform. When a small amount of TOP was added to the same solution and a change in the UV–vis absorption spectrum was noted (Figure S6). The antimony complex solution shows a clear broad absorption in the range from 325 to 425 nm, which is quenched by the addition of TOP. The disappearance of the absorption peak for antimony xanthate indicates that the complex decomposes in the presence of TOP. Similarly, when the xanthate complex of Ag was directly dispersed in TOP, due to its lack of solubility in organic solvents, it changed its color from greenish to brownish black, indicating the decomposition of the silver xanthate complex.

To examine the nature of the residual materials obtained after the dispersion of metal–organic complexes in TOP, the complexes (*c.a.* 0.1 g) were directly dispersed in TOP (3.0 mL) and sonicated for 15–20 min at room temperature. Afterward, acetone was added to precipitate the formed brownish-black residue. The removal of excess TOP, washing of the precipitate, and their separation were performed by centrifugation. The residual materials were dried and analyzed by IR and *p*-XRD analyses. The comparison between IR spectra of the complexes and the residual materials clearly shows the degradation of the complexes in the presence of TOP (Figure S7). The results indicate that TOP, besides acting as a capping agent, behaves as a decomposition initiator for Ag and Sb complexes and is sufficient for their reduction without any other reducing/capping agent.

TOP has been used earlier as a reducing agent, as trivalent phosphorus in TOP can be easily oxidized from a trivalent to a pentavalent state. Lee *et al.* converted functionalized graphene oxide to reduced graphene by using TOP, which was oxidized to trioctylphosphine oxide (TOPO).^32^ Mews *et al.* reported the synthesis of Bi nanoparticles by reduction of BiCl_3_ and Bi[N(SiMe_3_)_2_]_3_ precursors using only TOP, and ^31^P NMR spectrum confirmed the complete oxidation of TOP to TOPO.^33^ Furthermore, the comparative *p*-XRD analysis of the metal complexes and the residual materials also confirms the decomposition of the respective metal complexes in the presence of TOP (Figure S8). The diffraction pattern of silver xanthate residues indicates the formation of silver sulfide, whereas the decomposition of antimony xanthate by TOP yielded amorphous materials. Due to ambiguous diffraction patterns, it was difficult to confirm the formation of respective metal sulfide conclusively. However, the disappearance of diffraction peaks for respective xanthate complexes, together with UV–vis and IR spectra, confirms the decomposition of these complexes in TOP.

**Figure 3:**
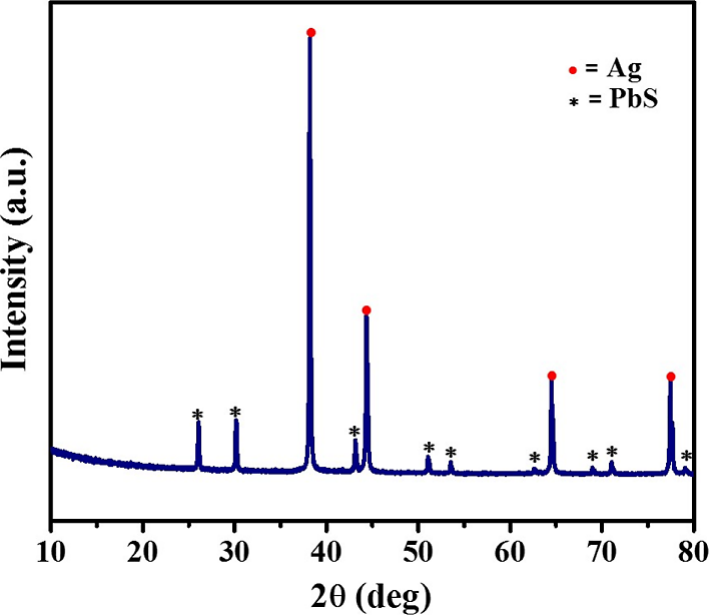
*p*-XRD analysis of the product obtained after the addition of lead nitrate to the reaction mixture.

The formation of metallic silver is either accompanied by the removal of sulfur as gaseous byproducts such as H_2_S or SO_2_ or an *in situ* reaction with TOP to form trioctylphosphine-sulfide (TOPS). In order to ascertain whether sulfur stayed in the solution or escaped as a gaseous byproduct, a small quantity of lead nitrate, dispersed in TOP, was added to the reaction mixture. The reaction was performed under similar conditions as for metallic silver. The diffraction pattern of the obtained product indicates that it consists of a mixture of Ag and PbS nanomaterials (Figure 3).

Interaction of phosphorus with sulfur atoms probably results in the oxidation of phosphorus from trivalent to pentavalent, *i.e.*, TOP to TOPS formation. At the same time, it reduces Ag^+^ to a zero-valent state, breaking the metal–sulfur bond, along with the formation of some other organic byproducts. The affinity of phosphorus for sulfur to form TOPS and three electron-donating alkyl chains attached to the phosphorus atom makes it a strong enough reductant for Ag^+^ to overcome its binding energy with the sulfide ion and convert it to the elemental state. The *in situ* formed TOPS reacts with Pb^2+^ ions to produce PbS nanoparticles. These results support the fact that sulfur is present in the solution.

Similarly, when antimony xanthate was decomposed in the presence of TOP, rhombohedral Sb (ICDD # 01-085-1322) was obtained (Figure S9). The sharpness and intensity of the diffraction peaks are an indication of the high crystallinity of the product, and no diffraction peaks corresponding to sulfide, oxide, or any other impurity are seen.

On the basis of these results, it can be inferred that the nature of the metal–organic precursors (*i.e.*, monovalent Ag xanthate or trivalent Sb xanthate) may not affect the reaction path, despite their different stability and decomposition mechanism. Obviously, the interesting question is whether this conclusion applies to the synthesis of bi-metallic/intermetallic alloyed nanomaterials using the same metal–organic precursors of Ag and Sb? Antimony forms an intermetallic phase compound with silver and is known as dyscrasite (Ag_3_Sb).^34^ Therefore, Ag and Sb xanthate complexes were mixed in a 3:1 ratio and the mixture was decomposed in the presence of TOP. Although the formation of a number of products (such as Ag, Sb, Ag_2_S, Sb_2_S_3_, or Sb doped Ag, and so on*.*) is possible, the *p*-XRD analysis confirms the formation of only dyscrasite phase (ICDD# 03-065-6359) (Figure 1d). Well-defined peaks with high intensity and sharpness were observed. The morphology of intermetallic nanoparticles, examined by TEM analysis, shows irregularly shaped particles that are larger in size than silver nanoparticles obtained by a similar route (Figure 2d). It was observed that particles containing antimony (*i.e.*, AgSbS_2_ and Ag_3_Sb) exhibit relatively more irregularity in a shape and size than Ag and Ag_2_S nanoparticles. The combination of different elements (Ag and Sb), coming from different precursors, may obviously affect the nucleation and growth of nanoparticles.

### Electrochemical Studies of Electrodes Modified with Obtained Ag-Based Nanomaterials

The electrochemical studies of electrodes modified with the prepared nanomaterials were focused on their redox and electrocatalytic activity toward ORR and H_2_O_2_. For this purpose, materials were trapped into a thin Nafion film deposited on the “noncatalytic” electrode surface.^35^ The ionomer network provided not only a scaffold for stable immobilization but also access to electrolytes and dissolved O_2_ to electrocatalytic sites. As we observed that the Sb and AgSbS_2_ nanoparticles were not stable and completely dissolved in the electrolyte under the applied (strongly alkaline medium and potential window) conditions, only Ag, Ag_3_Sb, and Ag_2_S nanoparticles were electrochemically studied.

**Figure 4:**
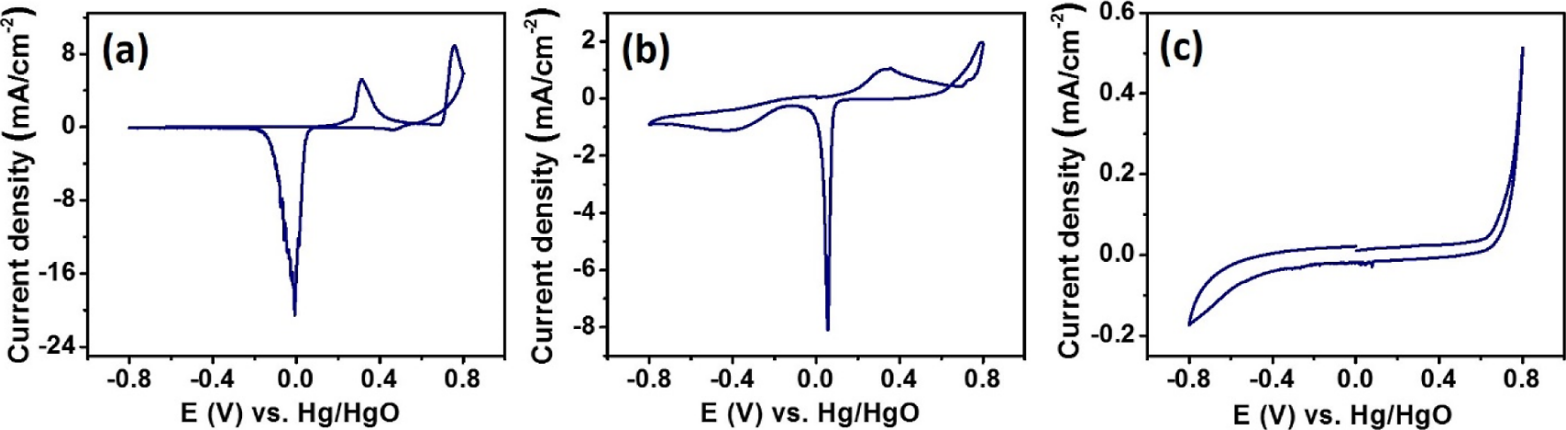
CVs obtained with the GC electrode modified with (a) Ag, (b) Ag_3_Sb, and (c) Ag_2_S nanoparticles, embedded in the Nafion film, in Ar-saturated 1 M KOH at 20 mV/s, [*E*(V) *vs* Hg/HgO].

First, cyclic voltammetry of Ag-, Ag_3_Sb-, and Ag_2_S-modified GCE was performed in an argon saturated solution. The set of asymmetric anodic and cathodic peaks seen on the voltammograms obtained with Ag and Ag_3_Sb (Figure 4) can be attributed to the oxidation of silver atoms to silver oxides, Ag_2_O and Ag_2_O_2_, and their re-reduction to metallic silver.^36^ The hysteresis seen at higher potentials may be connected with the passivation of the oxidation nanomaterial. The anodic and cathodic peak potentials are 0.043 and 0.049 V higher for Ag_3_Sb, which may indicate that Sb atoms slightly increase the stability of this phase. Significantly smaller peak currents (or charges) as compared to the electrode modified with pure Ag nanoparticles result from the fact that one out of four silver atoms is replaced by an antimony atom in the intermetallic compound. In contrast, the CV of Ag_2_S nanoparticle-modified electrode indicates no faradaic process within the accessible potential range defined by electrode reactions of the electrolyte. Perhaps the Ag–S bond is too strong to allow a change of Ag-oxidation state in the lattice structure. The sulfide ion is a soft base compared to an oxide ion (a hard base), and it binds much more strongly with a soft metal, *i.e.*, silver.

**Figure 5:**
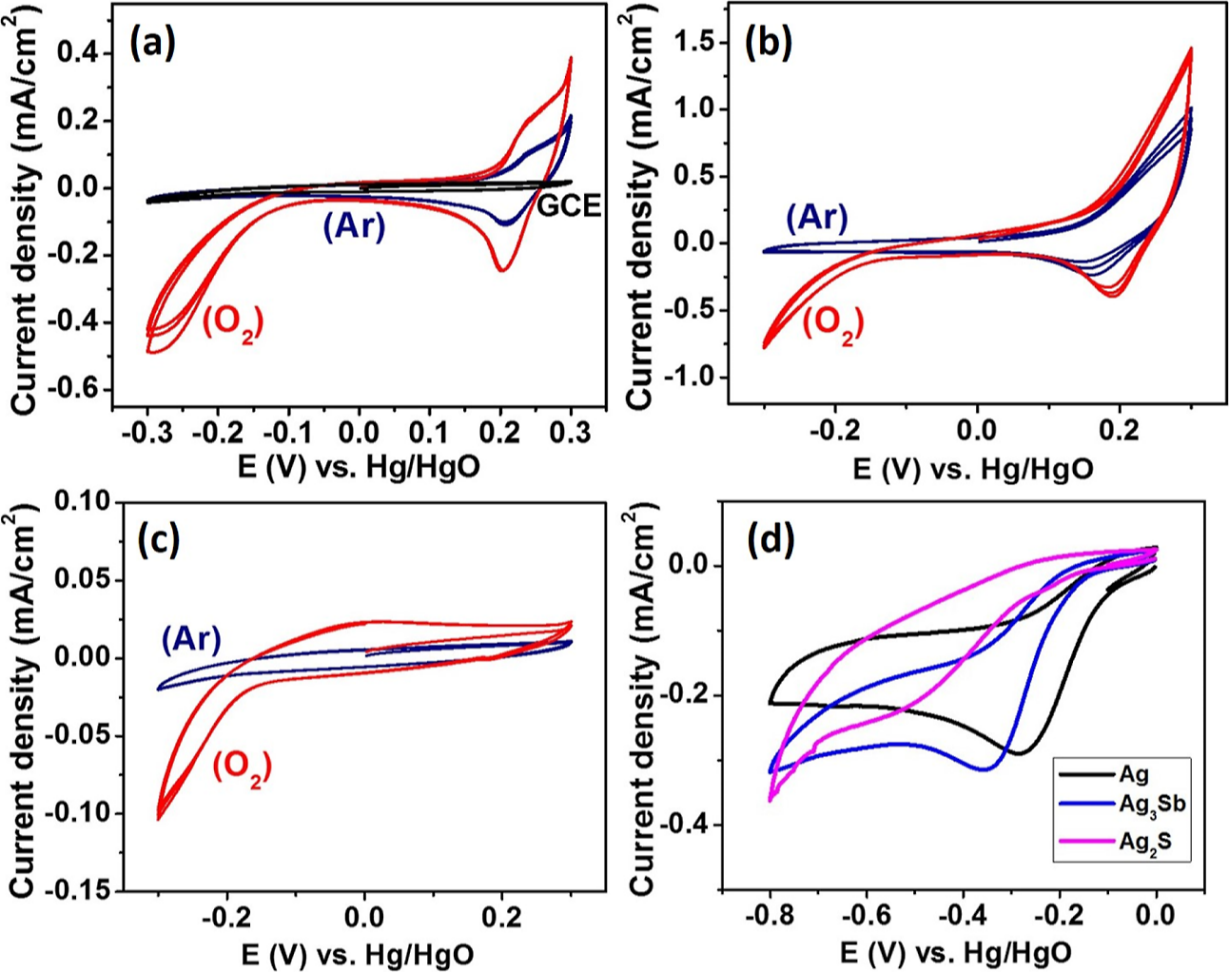
Comparison of voltammograms obtained with the GC electrode modified with (a) Ag, (b) Ag_3_Sb, and (c) Ag_2_S nanoparticles embedded in Nafion films in O_2_ (red curve) and Ar (black curve) saturated aqueous 1 M KOH. (d) Comparison of voltammograms obtained with the same electrodes in a different potential window in O_2_ saturated solution, The used electrocatalysts are marked in the figure. A scan rate of 20 mV/s.

Next, we focused on the electrocatalytic behavior of prepared nanomaterials. On the basis of the comparison of CVs of Ag, Ag_3_Sb, and Ag_2_S in oxygen- and argon-saturated alkaline electrolytes (Figure 5), one may conclude that cathodic current increases with onset potential −0.1―−0.2 V results from ORR. This current increase is not seen on unmodified GC electrodes indicating the electrocatalytic properties of the studied materials. After a few scans, the voltammograms become stable during subsequent scanning. The onset potential for Ag and Ag_3_Sb is similar (*c.a.* −0.1 V), whereas for Ag_2_S by *c.a.* 0.08 V lower. The set of peaks corresponding to the oxidation/reduction of silver is also seen on metal and intermetallic alloy-modified electrodes, and the presence of oxygen increases the magnitude of the peak currents.

Further excursion to lower potentials produces similar voltammograms for Ag- and Ag_3_Sb-modified electrodes with an onset potential lower by *c.a.* 0.07 V for the latter (Figure 5d). Their shape indicates diffusional control of ORR. This is not the case for the Ag_2_S-modified electrode, where the effect of sluggish kinetics can be seen with a similar onset potential as for the Ag_3_Sb-modified electrode. The other reason for lower ORR current recorded at Ag_2_S-modified electrodes may result from the fact that the electrocatalytic reaction occurs at the three-phase junction electrode|nanoparticle|electrolyte because of the insulating nature of this material. At conductive Ag or Ag_3_Sb nanoparticles, a reaction is expected to occur on their whole surface. Some decrease in the catalytic activity of all materials is seen during the first few consecutive scans (Figure S10). Clearly, the addition of a second component increases the onset potential as compared to that of the electrode modified with Ag nanoparticles prepared by the same method. It indicates that silver is the main component responsible for the oxygen reduction activity in the alloy. Perhaps the change of crystal lattice from face-centered cubic for Ag to orthorhombic (Ag_3_Sb) and monoclinic (Ag_2_S) and occupancy of different atoms (*i.e.*, Sb or S) alters the number of oxygen binding sites.

**Figure 6:**
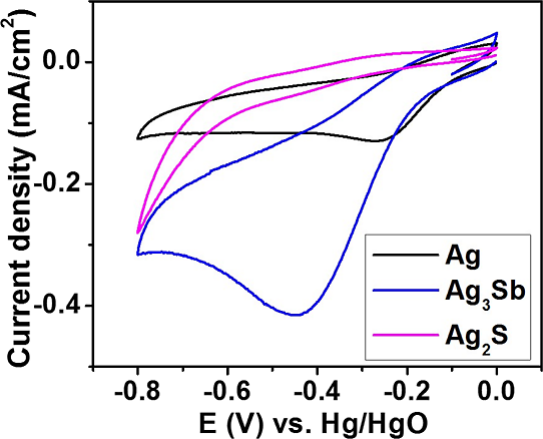
CV curves (first cycle) of all samples obtained in a 2.5 × 10^–5^ M H_2_O_2_ solution in 1 M aqueous KOH. Scan rate of 20 mV s^–1^. The used electrocatalysts are marked in the figure.

Because the formation of H_2_O_2_ is the first step of 4-electron ORR, its reduction was also studied. All modified electrodes exhibit a cathodic peak-shaped signal, which can be ascribed to this reaction (Figures 6 and S11). The onset potential at Ag- and Ag_3_Sb-modified electrodes differs by *c.a.* 0.02 V with a peak current 3 times higher for intermetallic alloy. This indicates that contrary to ORR, “dilution” of silver with antimony favors H_2_O_2_ reduction. Significantly, larger overpotential and lower current magnitude point out that Ag_2_S is a poor catalyst for this reaction as for ORR (see above).

### ORR at the Liquid–Liquid Interface

**Figure 7:**
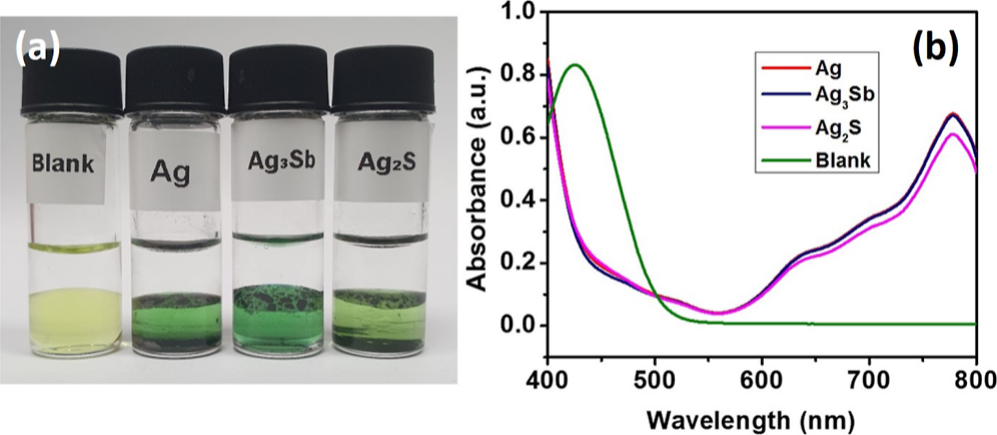
(a) Biphasic system consisting of 0.1 M aqueous HClO_4_ (top) and 5 mM DMFc in TFT solution (bottom) in the absence (blank) and presence of Ag, Ag_3_Sb, and Ag_2_S nanoparticles after 7 h and (b) UV–vis spectra of the organic phase collected from all experiments.

In order to get rid of the effect of electrode substrate and encapsulating polymer, ORR was also studied at the liquid|liquid interface. DMFc was used as an electron donor because of its sufficiently low redox potential to be an effective electron donor and hydrophobicity.^38^ Likewise, a polar highly hydrophobic organic solvent, such as dichloroethane (DCE), nitrobenzene, or TFT, are suitable immiscible organic solvents.^37^ TFT has been used as it is relatively less toxic. For this reaction, 1 mL of 5 mM DMFc solution in TFT was placed in a glass vial. Approximately, 2 mg of given nanoparticles were dispersed in 1 mL of 0.1 M aqueous HClO_4_ by sonication and this dispersion was placed over the organic phase. After a few minutes, the nanoparticles were dispersed in HClO_4_ assembled at the liquid–liquid interface. After 6 h, the color of the organic phase changed from yellow to green (Figure 7a), indicating the oxidation formation of the DMFc^+^ cation. One can also see that, at the same time, the color change is less intensive for the Ag_2_S sample. On the contrary, no significant change of color was observed in the absence of nanoparticles. DMFc oxidation was further confirmed by a change in UV–vis spectra (Figure 7b). The characteristic band for DMFc at 425 nm disappeared and the new one at 780 nm appeared with smaller intensity for the Ag_2_S sample, along with some shoulder peaks in the region of 600–800 nm. The control experiment almost does not change the spectrum. In order to confirm the formation of H_2_O_2_, a mixture of 0.1 M KI and 10% starch solution was added to the aqueous phase. The solution collected from the experiment with assembled Ag and Ag_2_S nanoparticles turned dark violet, whereas the sample collected from the experiment with Ag_3_Sb nanoparticles remained colorless (Figure S12). The violet color appears due to the formation of the I_3_-complex with starch in the presence of H_2_O_2_. It indicates that Ag and Ag_2_S nanoparticles assembled at the liquid|liquid interface facilitated the formation of H_2_O_2_ as an ORR product, as shown in eq 1:^37,38^
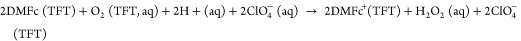
1

Because ORR was detected by the conversion of DMFc to DMFc^+^ cation by Ag_3_Sb, but H_2_O_2_ was not detected for this system *via* the KI-starch test, one may conclude that Ag_3_Sb catalyzes ORR *via* the 4-electron path, as shown in eq 2
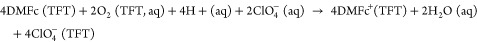
2

This result is consistent with the highest electrocatalytic activity of this nanomaterial toward H_2_O_2_ reduction seen in CV experiments.

To further verify the formation of H_2_O_2_ at the liquid|liquid interface and to make a quantitative estimation, SECM was used. For this purpose, CV curves were recorded in the potential range from −0.25―1.3 V *vs* Ag-wire quasi-reference electrode, at a scan rate of 100 mV s^–1^ at the SECM tip approaching the liquid|liquid interface in 20 μm steps. This procedure was applied to avoid the gradual loss of Pt activity toward the oxidation of H_2_O_2_.^39^ The approach curves were calculated from CV curves following the previously reported protocol.^23^ The oxidation current was recalculated to the H_2_O_2_ concentration.

**Figure 8:**
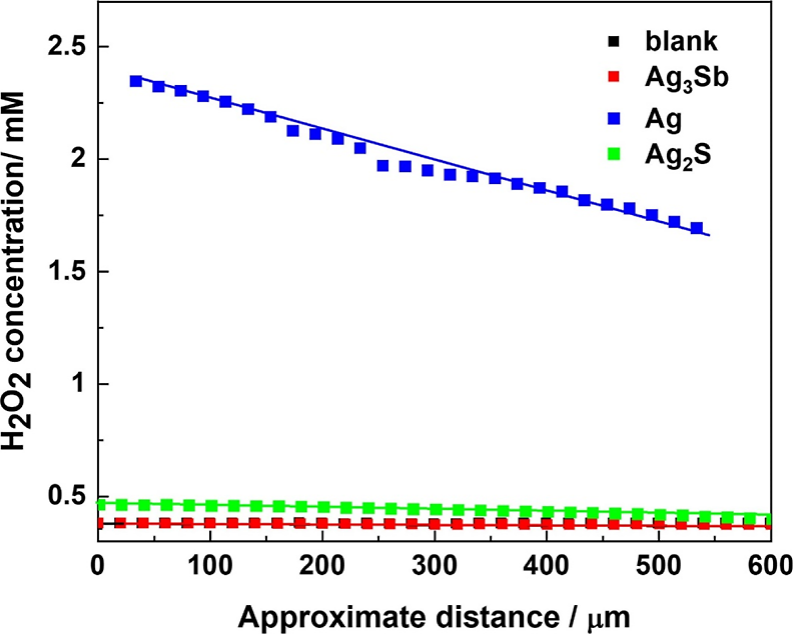
Distance-dependent H_2_O_2_ concentration obtained from SECM approach curves recorded in the absence of catalyst and the presence of Ag powder (blue), Ag_3_Sb powder (red), and Ag_2_S (green) in the 5 mM DMFc in the TFT/0.1 M HClO_4_ (aq) biphasic system. The SECM experiments were conducted 45 min after the preparation of the biphasic system.

Clearly, the closer the tip approaches the liquid|liquid interface, the larger the H_2_O_2_ concentration. At a given distance, the value of the latter depends on whether the catalyst is present at the interface and, more importantly, on the type of catalyst (Figure 8). The flux of H_2_O_2_ obtained from the slope of the approach curve for Ag_3_Sb (2.625 × 10^–12^ mol cm^–2^ s^–1^) was comparable to the blank, *i.e.*, without any catalyst at the interface, and negligible relative to the H_2_O_2_ flux for Ag (2.084 × 10^–10^ mol cm^–2^ s^–1^), which differs by more than 2 orders of magnitude (Figure 8). The H_2_O_2_ flux for Ag_2_S was also low (1.686 × 10^–11^ mol cm^–2^ s^–1^), which was anticipated due to the low activity demonstrated by its CV curves. Overall, the H_2_O_2_ flux increases in the order blank ≤ Ag_3_Sb < Ag_2_S < Ag. This SECM result obtained in the presence of Ag_3_Sb nanoparticles is consistent with the negative KI-starch test and supports the conclusion that in the presence of this catalyst, dioxygen is almost completely converted to H_2_O. Sb is relatively more oxophilic than Ag and, therefore, the presence of Sb may result in better adsorption of oxygen over the surface of the catalyst. The combined effect of reducing oxygen binding energy of Ag and increased oxygen adsorption affinity may have collectively facilitated the ORR *via* the 4-electron mechanism.

## Conclusions

We have demonstrated a new facile route for the synthesis of important elemental (Ag or Sb), binary metal sulfides (Ag_2_S and Sb_2_S_3_), intermetallic alloy (Ag_3_Sb), and ternary metal sulfide (AgSbS_2_) nanoparticles from simple molecular precursors (xanthate complexes) by controlling the reaction parameters. Previously, it has been shown that TOP can act as a phosphorus source and facilitate the formation of nickel phosphide nanomaterials.^40^ Here, we have demonstrated that TOP can also act as a reductant/desulfurizing agent and this property of TOP can be used to prepare bimetallic or intermetallic alloys.

The trends in the electrocatalytic performance of synthesized Ag-based nanomaterials (Ag_2_S, Ag_3_Sb, and Ag_2_S) were studied. These three catalysts provide an example of how their composition affects the ORR mechanism, which was proven consistently for the reaction at solid|liquid and liquid|liquid interfaces. The combination of an oxophilic atom with silver is probably suitable for ORR *via* the 4-electron mechanism. This, in turn, may help in the rational design of the Ag-based catalysts and a suitable combination with other elements.

## Supporting Information Available

*p*-XRD, TGA, UV spectra, IR spectra, CVs, and KI-starch test results (PDF)

## References

[ref1] aZaman S.; Huang L.; Douka A. I.; Yang H.; You B.; Xia B. Y. Oxygen reduction electrocatalysts toward practical fuel cells: progress and perspectives. Angew. Chem. 2021, 133, 17976–17996. 10.1002/ange.202016977. 33533165

[ref2] Liu J.; Gong Z.; Yan M.; He G.; Gong H.; Ye G.; Fei H. Electronic Structure Regulation of Single-Atom Catalysts for Electrochemical Oxygen Reduction to H_2_O_2_. Small 2022, 18, 2103824 10.1002/smll.202103824. 34729914

[ref3] Yang X.; Zeng Y.; Alnoush W.; Hou Y.; Higgins D.; Wu G. Tuning Two-Electron Oxygen-Reduction Pathways for H2O2 Electrosynthesis via Engineering Atomically Dispersed Single Metal Site Catalysts. Adv. Mater. 2022, 34, 2107954 10.1002/adma.202107954. 35133688

[ref4] Tang J.; Zhao T.; Solanki D.; Miao X.; Zhou W.; Hu S. Selective hydrogen peroxide conversion tailored by surface, interface, and device engineering. Joule 2021, 5, 1432–1461. 10.1016/j.joule.2021.04.012.

[ref5] aMiglbauer E.; Wójcik P. J.; Głowacki E. D. Single-compartment hydrogen peroxide fuel cells with poly (3, 4-ethylenedioxythiophene) cathodes. Chem. Commun. 2018, 54, 11873–11876. 10.1039/c8cc06802j. 30280179

[ref6] aLiu M.; Zhao Z.; Duan X.; Huang Y. Nanoscale structure design for high-performance Pt-based ORR catalysts. Adv. Mater. 2019, 31, 1802234 10.1002/adma.201802234. 30561854

[ref7] Erikson H.; Sarapuu A.; Tammeveski K. Oxygen reduction reaction on silver catalysts in alkaline media: a minireview. Chemelectrochem 2019, 6, 73–86. 10.1002/celc.201800913.

[ref8] aShumilova N.; Zhutaeva G.; Tarasevich M. Oxygen ionization on silver in alkaline solutions. Electrochim. Acta 1966, 11, 967–974. 10.1016/0013-4686(66)80035-x.

[ref9] Lima F.; De Castro J.; Ticianelli E. A. Silver-cobalt bimetallic particles for oxygen reduction in alkaline media. J. Power Sources 2006, 161, 806–812. 10.1016/j.jpowsour.2006.06.029.

[ref10] aLi L.; Wang P.; Shao Q.; Huang X. Recent progress in advanced electrocatalyst design for acidic oxygen evolution reaction. Adv. Mater. 2021, 33, 2004243 10.1002/adma.202004243. 33749035

[ref11] aWang Q.; Cui X.; Guan W.; Zhang L.; Fan X.; Shi Z.; Zheng W. Shape-dependent catalytic activity of oxygen reduction reaction (ORR) on silver nanodecahedra and nanocubes. J. Power Sources 2014, 269, 152–157. 10.1016/j.jpowsour.2014.06.160.

[ref12] aWu X.; Chen F.; Zhang N.; Lei Y.; Jin Y.; Qaseem A.; Johnston R. L. Activity trends of binary silver alloy nanocatalysts for oxygen reduction reaction in alkaline media. Small 2017, 13, 1603387 10.1002/smll.201603387. 28151572

[ref13] aTran H. V.; Le T. A.; Giang B. L.; Piro B.; Tran L. D. Silver nanoparticles on graphene quantum dots as nanozyme for efficient H2O2 reduction in a glucose biosensor. Mater. Res. Express 2019, 6, 115403 10.1088/2053-1591/ab46ca.

[ref14] aBetancourt L. E.; Rojas-Perez A.; Orozco I.; Frenkel A. I.; Li Y.; Sasaki K.; Senanayake S. D.; Cabrera C. R. Enhancing ORR performance of bimetallic PdAg electrocatalysts by designing interactions between Pd and Ag. ACS Appl. Energy Mater. 2020, 3, 2342–2349. 10.1021/acsaem.9b01920.

[ref15] Wang Q.; Chen F.; Liu Y.; Zhang N.; An L.; Johnston R. L. Bifunctional electrocatalysts for oxygen reduction and borohydride oxidation reactions using Ag3Sn nanointermetallic for the ensemble effect. ACS Appl. Mater. Interfaces 2017, 9, 35701–35711. 10.1021/acsami.7b05186. 28953357

[ref16] Lu Y.; Zhang N.; An L.; Li X.; Xia D. Synthesis of high dispersed intermetallic Ag_4_Sn/C and its enhanced oxygen reduction reaction activity. J. Power Sources 2013, 240, 606–611. 10.1016/j.jpowsour.2013.05.015.

[ref17] Gonen S.; Lori O.; Fleker O.; Elbaz L. Electrocatalytically Active Silver Organic Framework: Ag (I)-Complex Incorporated in Activated Carbon. ChemCatChem 2019, 11, 6124–6130. 10.1002/cctc.201901604.

[ref18] Khan M. D.; Malik M. A.; Revaprasadu N. Progress in selenium based metal-organic precursors for main group and transition metal selenide thin films and nanomaterials. Coord. Chem. Rev. 2019, 388, 24–47. 10.1016/j.ccr.2019.02.026.

[ref19] Sarker J. C.; Hogarth G. Dithiocarbamate complexes as single source precursors to nanoscale binary, ternary and quaternary metal sulfides. Chem. Rev. 2021, 121, 6057–6123. 10.1021/acs.chemrev.0c01183. 33847480

[ref20] aPeljo P.; Scanlon M. D.; Olaya A. J.; Rivier L.; Smirnov E.; Girault H. H. Redox electrocatalysis of floating nanoparticles: determining electrocatalytic properties without the influence of solid supports. J. Phys. Chem. Lett. 2017, 8, 3564–3575. 10.1021/acs.jpclett.7b00685. 28707892

[ref21] aSmirnov E.; Peljo P.; Scanlon M. D.; Girault H. H. Gold nanofilm redox catalysis for oxygen reduction at soft interfaces. Electrochim. Acta 2016, 197, 362–373. 10.1016/j.electacta.2015.10.104.

[ref22] Jedraszko J.; Krysiak O.; Adamiak W.; Nogala W.; Girault H. H.; Opallo M. H_2_O_2_ Generation at a Carbon-Paste Electrode with Decamethylferrocene in 2-Nitrophenyloctyl Ether as a Binder: Catalytic Effect of MoS2 Particles. Chemelectrochem 2016, 3, 1400–1406. 10.1002/celc.201600242.

[ref23] Warczak M.; Osial M.; Urbanska W.; Pisarek M.; Nogala W.; Opallo M. Hydrogen peroxide generation catalyzed by battery waste material. Electrochem. Commun. 2022, 136, 107239 10.1016/j.elecom.2022.107239.

[ref24] aAlqahtani T.; Khan M. D.; Kelly D. J.; Haigh S. J.; Lewis D. J.; O’Brien P. Synthesis of Bi 2– 2x Sb 2x S 3 (0≤ x≤ 1) solid solutions from solventless thermolysis of metal xanthate precursors. J. Mater. Chem. C 2018, 6, 12652–12659. 10.1039/c8tc02374c.

[ref25] aAlqahtani T.; Khan M. D.; Lewis D. J.; Zhong X. L.; O’Brien P. Scalable synthesis of Cu–Sb–S phases from reactive melts of metal xanthates and effect of cationic manipulation on structural and optical properties. Sci. Rep. 2021, 11, 1887 10.1038/s41598-020-80951-5. 33479247 PMC7820284

[ref26] aKhan M.; Malik M.; Akhtar J.; Mlowe S.; Revaprasadu N. Phase pure deposition of flower-like thin films by aerosol assisted chemical vapor deposition and solvent mediated structural transformation in copper sulfide nanostructures. Thin Solid Films 2017, 638, 338–344. 10.1016/j.tsf.2017.07.064.

[ref27] Steggerda J.; Cras J.; Willemse J. Reactions of complexes of dithiocarbamate and related ligands. Recl. Trav. Chim. Pays-Bas 2010, 100, 41–48. 10.1002/recl.19811000202.

[ref28] Hollingsworth N.; Roffey A.; Islam H.-U.; Mercy M.; Roldan A.; Bras W.; Wolthers M.; Catlow C. R. A.; Sankar G.; Hogarth G.; et al. Active nature of primary amines during thermal decomposition of nickel dithiocarbamates to nickel sulfide nanoparticles. Chem. Mater. 2014, 26, 6281–6292. 10.1021/cm503174z.

[ref29] Pearson R. G. Hard and soft acids and bases. J. Am. Chem. Soc. 1963, 85, 3533–3539. 10.1021/ja00905a001.

[ref30] aEensalu J. S.; Tonsuaadu K.; Oja Acik I.; Krunks M. Sb2S3 thin films by ultrasonic spray pyrolysis of antimony ethyl xanthate. Mater. Sci. Semicond. Process. 2022, 137, 106209 10.1016/j.mssp.2021.106209.

[ref31] Vakalopoulou E.; Knez D.; Sigl M.; Kothleitner G.; Trimmel G.; Rath T. A Colloidal Synthesis Route Towards AgBiS_2_ Nanocrystals Based on Metal Xanthate Precursors. ChemNanoMat 2022, 9, e202200414 10.1002/cnma.202200414.

[ref32] Liu J.; Jeong H.; Liu J.; Lee K.; Park J.-Y.; Ahn Y.; Lee S. Reduction of functionalized graphite oxides by trioctylphosphine in non-polar organic solvents. Carbon 2010, 48, 2282–2289. 10.1016/j.carbon.2010.03.002.

[ref33] Li Z.; Kornowski A.; Myalitsin A.; Mews A. Formation and function of bismuth nanocatalysts for the solution–liquid–solid synthesis of CdSe nanowires. Small 2008, 4, 1698–1702. 10.1002/smll.200800858. 18780365

[ref34] Scott J. D. Refinement of the crystal structure of dyscrasite, and its implications for the structure of allargentum. Can. Mineral. 1976, 14, 139–142.

[ref35] Liu H.-Y.; Anson F. C. Redox mediation of dioxygen reduction within Nafion electrode coatings containing colloidal platinum as catalyst. J. Electroanal. Chem. Interfacial Electrochem. 1983, 158, 181–185. 10.1016/0022-0728(83)80363-5.

[ref36] aGuo J.; Hsu A.; Chu D.; Chen R. Improving oxygen reduction reaction activities on carbon-supported Ag nanoparticles in alkaline solutions. J. Phys. Chem. C 2010, 114, 4324–4330. 10.1021/jp910790u.

[ref37] Adamiak W.; Jedraszko J.; Nogala W.; Jönsson-Niedziolka M.; Dongmo S.; Wittstock G.; Girault H. H.; Opallo M. A simple liquid–liquid biphasic system for hydrogen peroxide generation. J. Phys. Chem. C 2015, 119, 20011–20015. 10.1021/acs.jpcc.5b06620.

[ref38] Su B.; Nia R. P.; Li F.; Hojeij M.; Prudent M.; Corminboeuf C.; Samec Z.; Girault H. H. H2O2 generation by decamethylferrocene at a liquid| liquid interface. Angew. Chem. 2008, 120, 4753–4756. 10.1002/ange.200801004. 18484580

[ref39] Evans S. A.; Elliott J. M.; Andrews L. M.; Bartlett P. N.; Doyle P. J.; Denuault G. Detection of hydrogen peroxide at mesoporous platinum microelectrodes. Anal. Chem. 2002, 74, 1322–1326. 10.1021/ac011052p. 11924592

[ref40] aAyom G. E.; Khan M. D.; Ingsel T.; Lin W.; Gupta R. K.; Zamisa S. J.; Zyl W. E.; Revaprasadu N. Flexible molecular precursors for selective decomposition to nickel sulfide or nickel phosphide for water splitting and supercapacitance. Chem.—Eur J. 2020, 26, 2693–2704. 10.1002/chem.201904583. 31773811

