# Supplementary material for: Molecular Precursor Routes for Ag-Based Metallic, Intermetallic, and Metal Sulfide Nanoparticles: Their Comparative ORR Activity Trend at Solid|Liquid and Liquid|Liquid Interfaces
Source: Inorg Chem. 2023 May 16;62(21):8379–88. doi: 10.1021/acs.inorgchem.3c00978 (PMC10230501; doi:10.1021/acs.inorgchem.3c00978)
Supplement: Supplementary file 1 — ic3c00978_si_001.pdf [file ic3c00978_si_001.pdf]

Supplementary Data

Molecular precursor route for Ag-based metallic, intermetallic and metal sulfide nanoparticles: Their comparative ORR activity trend at solid|liquid and liquid|liquid interface

Malik Dilshad Khan,[1,4*] Magdalena Warczak,[1,2] Ginena Bildard Shombe,[3,4] Neerish Revaprasadu[4] and Marcin Opallo[1*]

[1]Institute of Physical Chemistry, Polish Academy of Sciences, Kasprzaka 44/52, 01-224 Warsaw, Poland.
[2]Department of Food Analysis and Environmental Protection, Faculty of Chemical Technology and Engineering, Bydgoszcz University of Science and Technology, Seminaryjna 3, 85-326 Bydgoszcz, Poland.
[3]Chemistry Department, University of Dar-es-Salaam, P.O. Box 35061, Dar-es-Salaam, Tanzania.
[4]Department of Chemistry, University of Zululand, Private bag X1001, Kwa-Dlangezwa 3880, South Africa.

*E-mail: malikdilshad@ichf.edu.pl ; mopallo@ichf.edu.pl

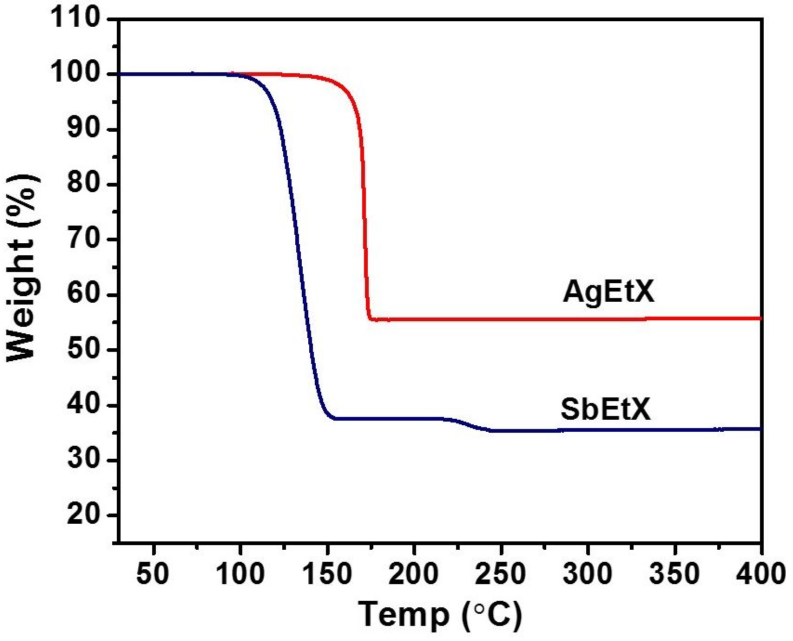

Figure S1. Thermogravimetric analysis of antimony and silver xanthate complexes, respectively.

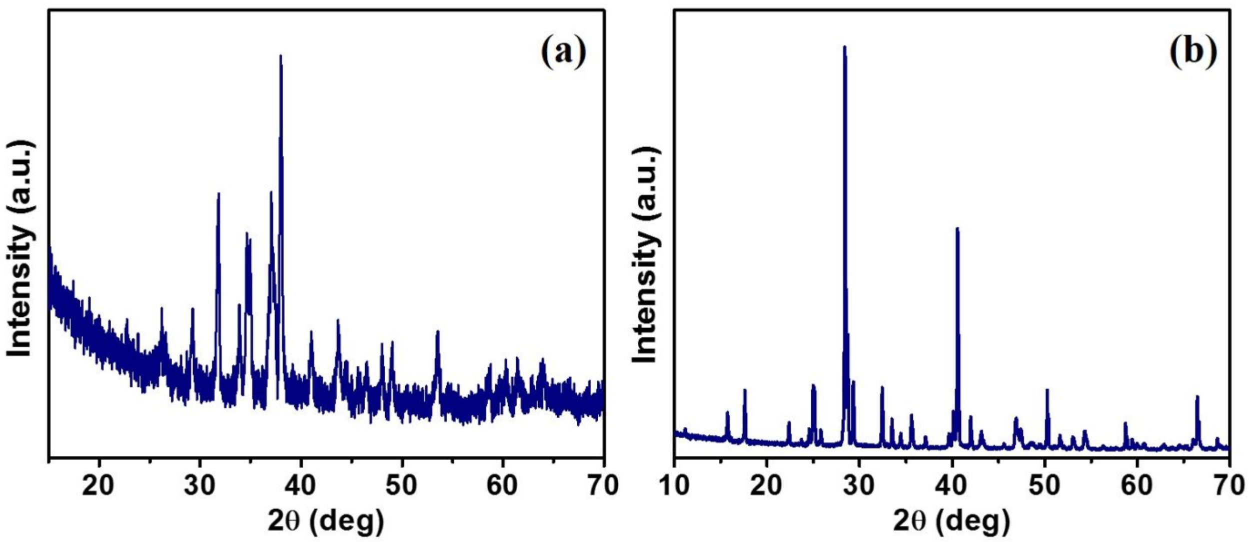

Figure S2: p-XRD of products obtained after solid-state decomposition of (a) silver xanthate and (b) antimony xanthate complexes under an inert environment.

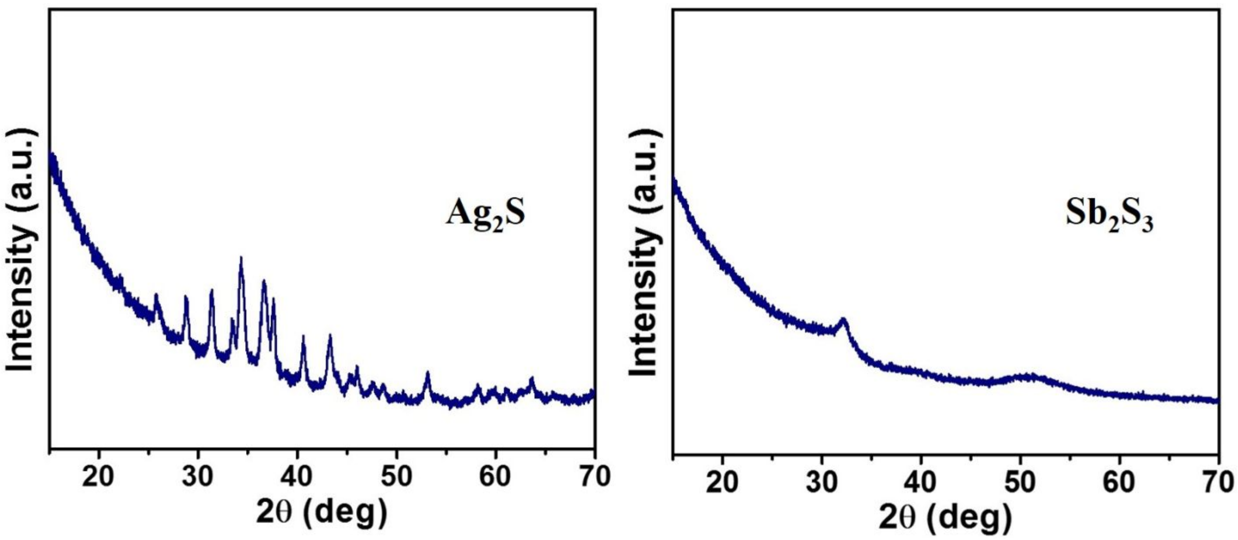

Figure S3: p-XRD analysis of decomposition products of Ag and Sb xanthate in oleylamine at room temperature. Ag xanthate yielded Ag_2S with well-defined diffraction peaks, whereas the decomposition of Sb xanthate yielded amorphous Sb_2S_3.

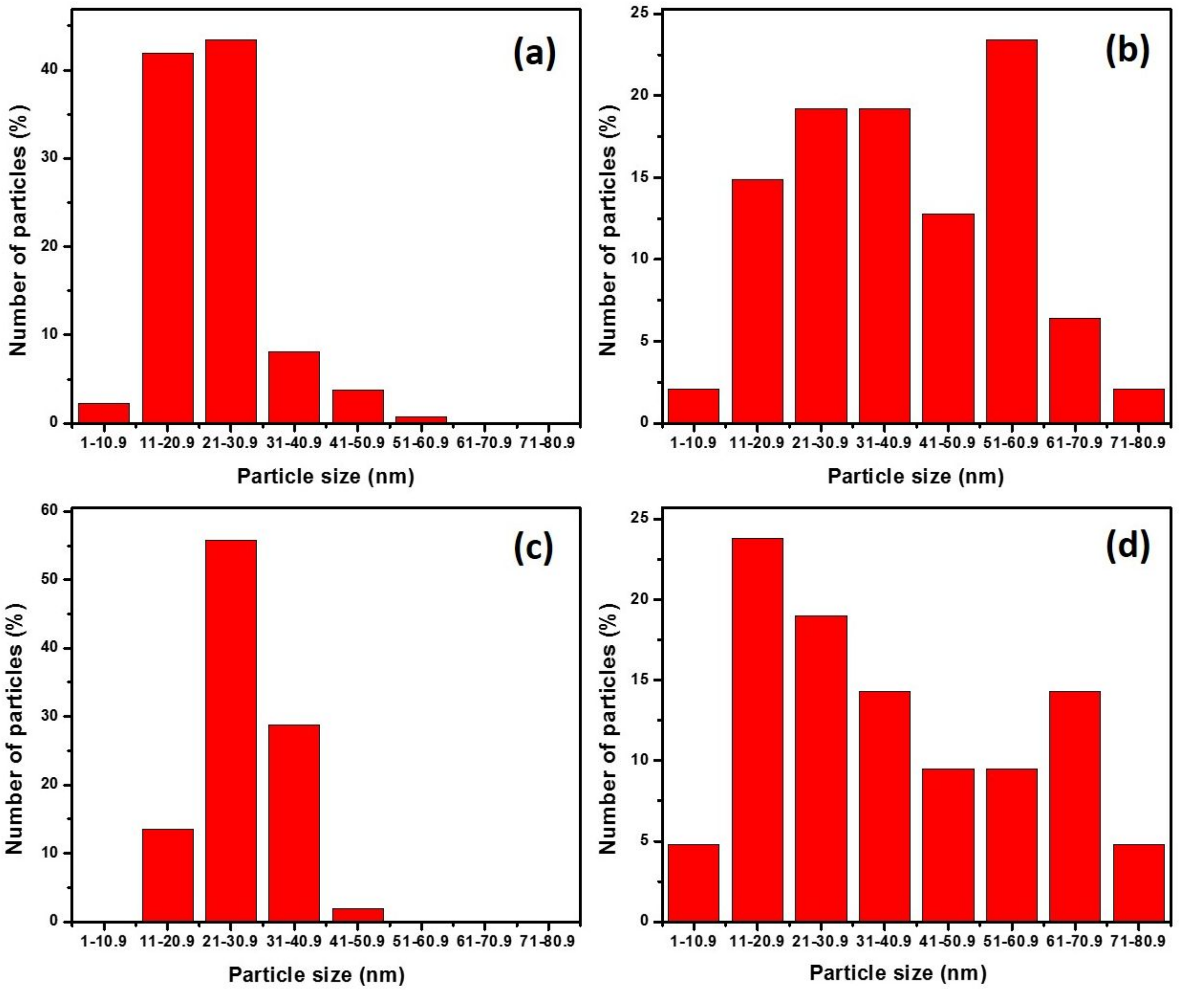

Figure S4: Size distribution histogram for (a) Ag_2S, (b) $AgSbS_2$, (c) Ag and (d) Ag_3Sb nanomaterials synthesized in oleylamine at 200 °C.

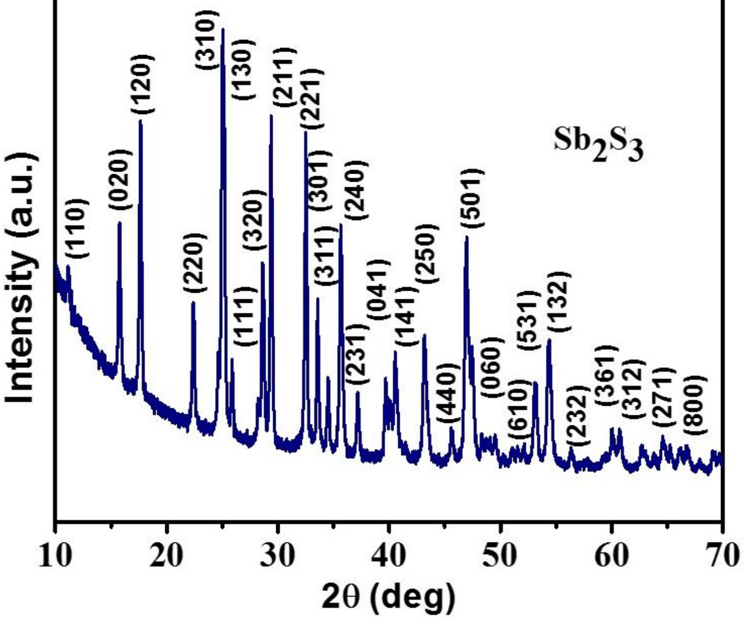

Figure S5. P-XRD analysis of Sb_2S_3 synthesized by the decomposition of antimony xanthate complex in oleylamine at 200 °C, when 1-ODE is used as a dispersion medium.

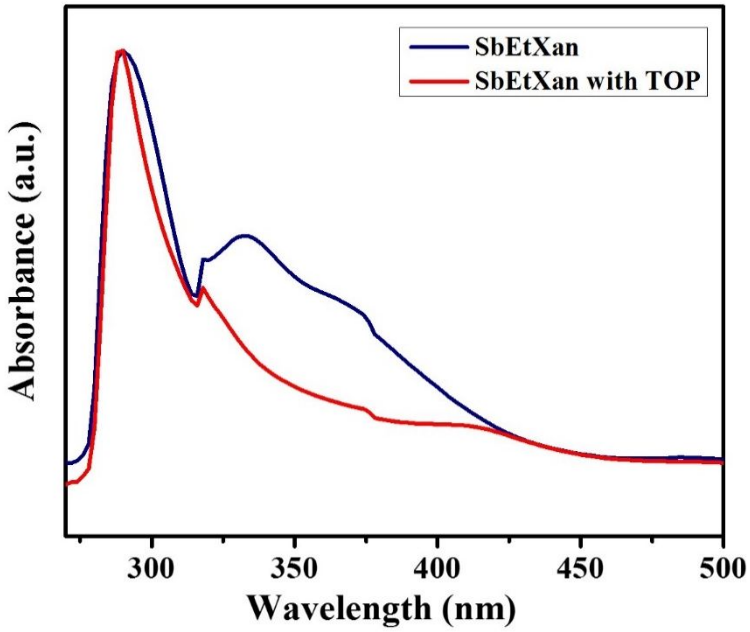

Figure S6. UV-Vis spectra of antimony ethyl xanthate (SbEtXan), dissolved in chloroform, in the absence and presence of trioctylphosphine (TOP).

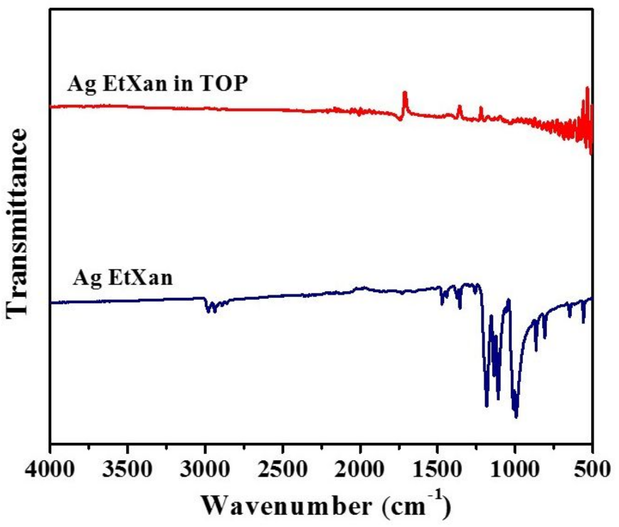

Figure S7. IR spectra of powdered antimony and silver xanthate complexes before and after the addition of TOP.

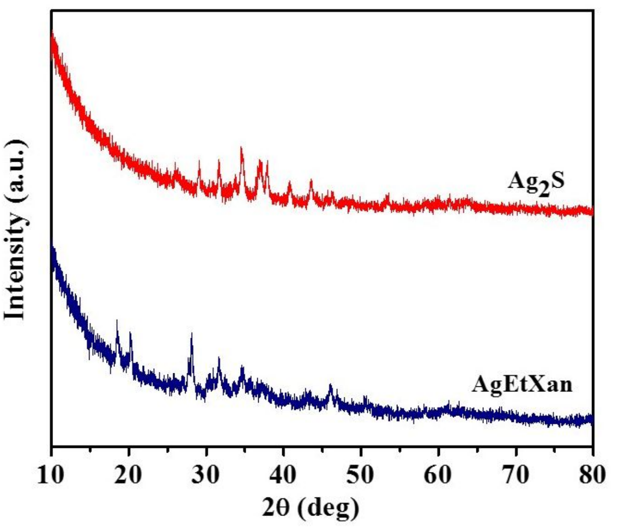

Figure S8. P-XRD patterns of antimony and silver xanthate complexes and the residue obtained after the addition of TOP.

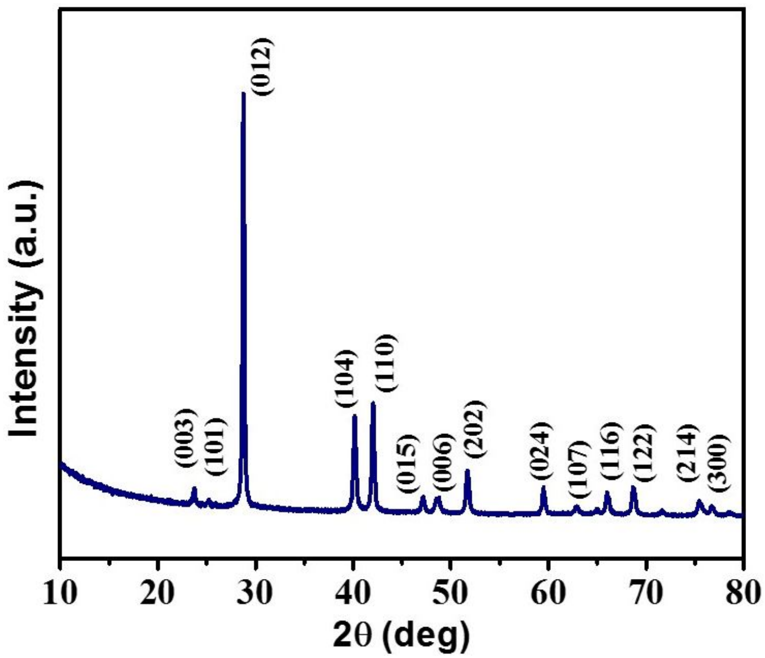

Figure S9. P-XRD analysis of Sb synthesized by the decomposition of antimony xanthate complex in oleylamine at 200 °C, when TOP is used as a dispersion medium.

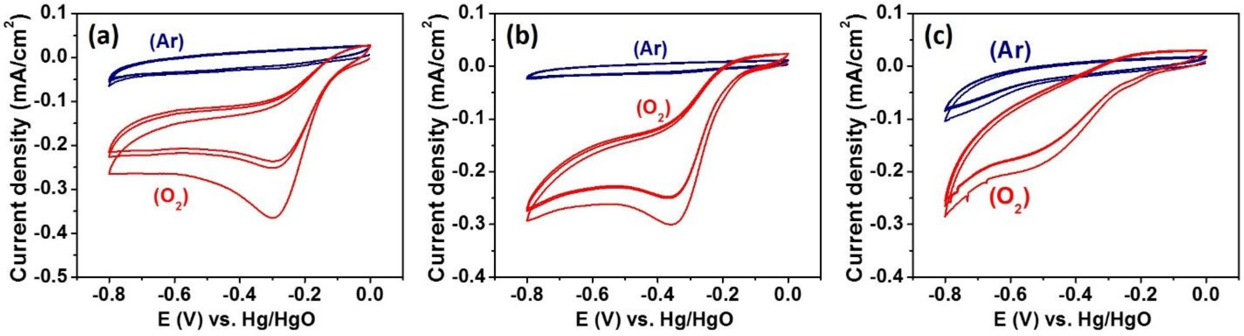

Figure S10. Subsequent first cyclic voltammograms obtained with a GC electrode modified with (a) Ag, (b) Ag_3Sb, and (c) Ag_2S nanoparticles embedded in Nafion film, in Ar and O_2 saturated 1M aqueous KOH. Scan rate 20 mV/s.

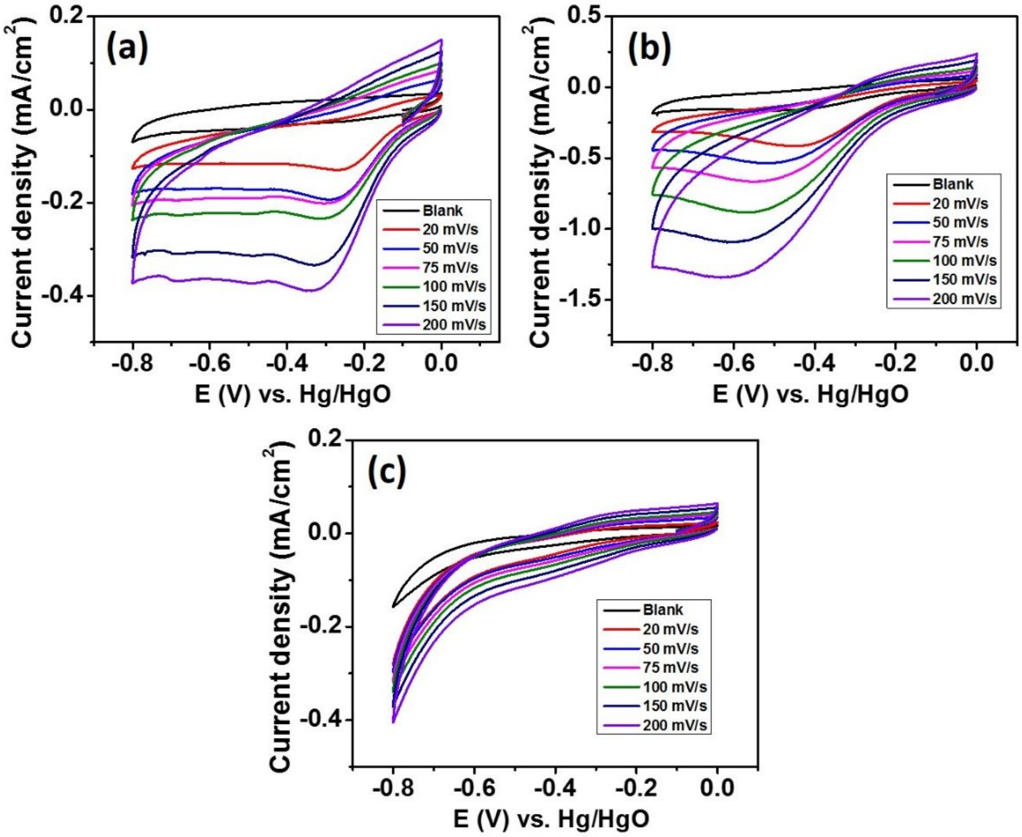

Figure S11. The effect of scan rate on CV curves of all samples obtained at GC electrode modified with Ag, Ag_3Sb, and Ag_2S in 2.5×10^{-5} M H_2O_2 solution in 1 M KOH. CV curve for bare GC electrode is represented as blank.

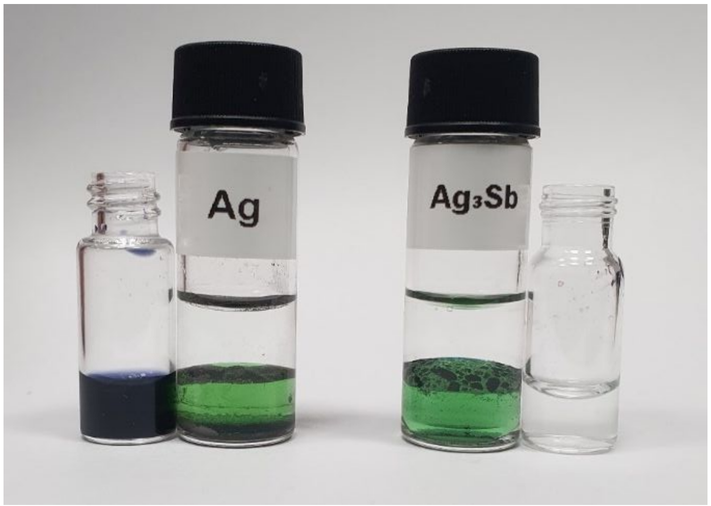

Figure S12. Result of KI-starch test for the detection of H_2O_2 in the aqueous phase collected from the biphasic system with Ag (left) and Ag_3Sb (right) nanoparticles.
